# Supplementary material for: Are carbon emissions trading and green financial instruments synergistic? -Comprehensive quantitative research based on content analysis
Source: PLoS One. 2024 Mar 7;19(3):e0298601. doi: 10.1371/journal.pone.0298601 (PMC10919601; doi:10.1371/journal.pone.0298601)
Supplement: S1 Data — (ZIP) [file pone.0298601.s001.zip › Data/地级市Z_F工作报告.zip!/─■╧─/╩»╫∞╔╜╩╨/└┤╫╘╣1⁄2╓┌║┼í╛┬φ┐╦╩2╛▌═°í┐_2016.docx]

各位代表：

现在，我代表市人民政府，向大会作政府工作报告，请各位代表连同《石嘴山市国民经济和社会发展第十三个五年规划纲要(草案)》一并审议，并请市政协委员和列席人员提出意见。

一、2015年及“十二五”工作回顾

2015年，宏观经济形势更加严峻复杂。面对前所未有的压力和挑战，在自治区党委、政府和市委的坚强领导下，我们积极适应经济发展新常态，围绕市十三届人大三次会议确定的目标任务，主动作为，奋力攻坚，统筹做好稳增长、调结构、促改革、惠民生、防风险各项工作，经济社会发展总体平稳，稳中有进。全年实现地区生产总值482.4亿元，增长6.9%;固定资产投资498.9亿元，增长0.2%;一般公共财政预算收入26.8亿元，可比增长6%;城镇和农村居民人均可支配收入分别达到24168元、10995元，增长8%和7.6%。

一年来，我们主要做了以下工作：

(一)综合施策保增长，经济运行稳中趋好。完善市级领导包抓企业、包推项目责任制，每月对经济运行进行分析研判，确定月度调控重点，积极应对经济下行压力。落实自治区新“工业18条”，出台工业扩增抑减促复产7条措施，一企一策帮助解决难题。协调金融机构向83户企业授信31亿元，3家企业发行债券融资21亿元，7家企业在新三板、上海股交中心挂牌交易。政银企联动，化解了部分企业出现的资金链、担保链风险。新增规模以上企业23家，规上工业增加值增长7.3%;重点项目开工117个，开工率96%，完成投资220亿元;120个项目列入自治区重大工程项目包，12个项目列入国家发改委PPP项目库，37个项目获国家15.4亿元专项建设债券基金支持;争取各类资金79.2亿元，同比增长12.4%;争取先行先试政策，列入全国中小城市综合改革试点、全国首批15个小微企业创业创新基地示范城市、国家循环经济示范城市、国家生态文明先行示范区、中国科协创新驱动助力工程示范市。精心编制“十三五”规划纲要，谋划储备项目129个，估算总投资3200多亿元。

(二)多措并举促转型，产业升级步伐加快。建立产业转型升级统计监测体系，设立产业发展引导基金，加快产业结构调整，第三产业比重提高0.8个百分点，非高载能产业占规上工业比重提高1.3个百分点，轻工业生产和效益“双增长”。狠抓工业项目不放松，大地轮胎一期、恒达纺织一期、中利电缆一期等20 个项目建成投产，实施164个循环化改造和工业技改项目，推进15家企业炉型置换和烟气余热综合利用，淘汰落后产能79.4万吨。与兰州铁路局签署合作协议，开通“陆港号”集装箱专列，物流业完成货运量同比增长15%;环星海湖开发有序推进，奇石山文化旅游城、海成·星湖天地等项目进展顺利;建成汉唐商业街一期、万德隆商业广场等4个特色商业街区;开通沙湖名优产品展示馆等电子商务平台。加快推进国家农业科技园区建设，新培育农业产业化龙头企业22家、家庭农场68家，建成粮食高产示范区12个、各类特色种养示范园区154个，粮食生产实现“十二连增”，“一优五特”产业稳步壮大。

(三)坚持不懈抓统筹，城乡建设协调推进。扎实开展“三规合一”及“多规融合”试点，形成了较为完善的规划体系。改造老旧住宅小区35.6万平米，创建规范化物业服务小区20个，建成公交始末站4个、港湾式公交站亭110个。建设美丽小城镇3个、美丽乡村16个。石嘴山黄河公路大桥、金水路连接线、五岳路延伸段建成通车。建设市民休闲森林公园3个，新增绿化面积4万亩，全面完成主干道路大整治大绿化。实施银北百万亩盐碱地改良工程，改造中低产田 20.9万亩，新增节水灌溉面积12万亩。完成柳条沟、大峰沟等山洪沟道治理，城市防洪标准进一步提高。开展环境保护执法年活动，推进环保“三项行动”和 “六项”治理，取缔淘汰城市建成区燃煤茶浴炉187台，PM10同比下降3.9%，环境空气质量优良天数达到228天。

(四)借助外力促发展，对外开放不断深化。与黎巴嫩迪琨尼市和乌海市、鄂尔多斯市等缔结为友好城市。组团参加了“海峡两岸(宁夏)经贸文化合作交流活动周”，在台中举办了宁夏石嘴山两岸产业合作交流会，搭建了与台湾企业合作新平台。深化与中建材、中煤、中钢等央企合作，与上海电气签署战略合作协议，与中国通用合作建设开发区污水处理等项目。加大产业项目招商力度，与杉杉能源签订建设10万吨锂电池正极材料协议，引进恒达纺织、大唐火电等40个重大项目，招商引资实际到位资金350亿元。着力打造开放平台，争取国家、自治区资金1.4亿元，市、县区投入8000万元，撬动社会投资4亿元，加强开发区基础设施建设。成立了“中关村天合科技成果转化石嘴山分中心”。借力中国科协“连线”，50家全国学会、120位专家与我市80多家重点企业对接，在人才培训、新产品研发、科技成果转化等方面提供支持。召开全市科技创新奖励大会，对52项科技创新项目进行了表彰奖励。承办宁夏高技能人才发展论坛，为中德高技能人才培养示范基地挂牌，宁夏理工学院与美国西北理工大学、东北大学等合作办学不断深入。举办了国际铁人三项赛、全国室内五人制足球赛、首届国际标准舞公开赛、星海湖户外运动嘉年华等活动，提高了对外知名度和影响力。

(五)务实创新抓改革，发展活力明显增强。深化行政审批制度改革，被列入自治区建设项目“多规合一”并联审批试点;取消、降低和免征行政事业性收费33项，降低和暂停中介服务收费9项，放开了53项价格及收费管理。推进商事制度改革,实行“三证合一”、“一照一码”登记制度，全市登记注册小微企业同比增长22.5%，登记个体工商户同比增长3.7%。稳步推进市属国有企业改革，完成一些部门政企分离脱钩改制，组建2家混合所有制企业。加快招投标制度改革，对500万元以下的政府投资项目实行随机抽取定标办法。农村土地经营管理制度改革向纵深推进，办理农村土地经营权证贷款2.3亿元，落实农民“三权”自愿有偿转让1300户、6600亩，全市流转土地达到57.9万亩;启动实施农村产权流转交易市场、宅基地制度改革等试点工作。

(六)加大投入惠民生，社会事业全面进步。在财政增收困难的情况下，将72%的财力用于保障改善民生，10件民生实事全部完成。学前教育三年入园率高于全区平均水平18个百分点，在全国率先实现县区义务教育基本均衡发展目标。举办了首届市民运动会，代表宁夏参加全国第九届残运会暨第六届特殊奥林匹克运动会并获得55枚奖牌，参加全区第八届少数民族传统体育运动会，取得了总分第三、五市第二的好成绩。推进县级公立医院改革，实行“先住院后付费”诊疗模式，取消全部药品加成。成功创建首批全国幸福家庭活动示范市。开展新型职业农民和农村劳动力职业技能培训4319人，扶持157名大学生自主创业，全市新增城镇就业1.94万人，登记失业率为3.9%。建成失智失能护理院、永峰养老院等4家养老服务机构。建设棚户区安置房8815套、公共租赁住房2563 套。推动国家公共文化服务体系示范区创建，整合57项文化体育资源，免费向群众开放。“六五”普法通过国家验收，建成42家法律服务超市，为困难群众免费办理法律援助案件1219件，挽回经济损失4057万元。统筹抓好平安建设等工作，刑事案件总量同比下降6.1%，安全生产事故起数和死亡人数实现“双下降”。

(七)持之以恒转作风，行政效能有效提高。扎实开展 “三严三实”专题教育，持续整治“四风”，对2013年以前遗留的67个重点问题进行梳理，集中力量解决了惠农河滨街居民供暖、大武口星海时代小区旧楼拆迁等32个问题。健全行政决策机制，完善政府法律顾问团制度，对128件政府重大决策进行法律审查，依法行政能力得到提升。强化部门平时监督，实行按季考评，测评结果在新闻媒体公开。率先将第三方评估引入人大代表建议和政协委员提案办理工作，办理市人大代表建议64件、政协委员提案163件。多措并举化解信访事项，信访总量同比下降36.1%，全区信访工作规范化建设推进会在我市召开。强化审计监督，核减各类工程项目资金5640万元。畅通问政渠道，办理市长信访批示件175件，办结率92%。举办“电视问政”4期，推动解决了道路安全、噪音扰民等137项群众反映的热点、难点问题。开展明察暗访62批次，加大问责力度，政风行风明显好转。

各位代表，总结2015年，我们深感过程异常艰辛，成绩来之不易。回顾“十二五”，我们遇到的困难前所未有,付出的努力非同寻常,取得的成果令人欣慰。可以说，“十二五”时期是我市发展极不平凡的五年。五年来，在经济下行压力持续加大、结构性矛盾突出等诸多不利因素的影响下，全市上下负重拼搏、开拓创新、攻坚克难，大力实施工业强市、民生优先、城乡统筹、生态立市、人才支撑“五大战略”，推进工业升级、三产扩张、民生幸福、环境改善、城乡统筹、社会管理“六项攻坚工程”，推动产业、民生、生态“三大转型”，呈现出经济平稳增长、民生持续改善、社会和谐稳定的良好局面。综合实力显著增强。全市地区生产总值(GDP)达到482.4亿元，年均增长9.8%，高于全国平均水平2个百分点，人均GDP达到62650元，居五市第一。五年累计完成固定资产投资 2135亿元，年均增长13.1%;完成地方财政收入211亿元，较“十一五”时期增长2倍多。社会消费品零售总额达到96亿元，年均增长9.3%。产业层次明显提升。争取并实施全国老工业基地调整改造、国家承接产业转移示范区、国家公共文化服务体系示范区等政策支持，建成大地循环、申银特钢、丽珠医药等一批大项目。突出抓好产业转型升级和结构调整，工业“4+4”产业初具规模，非煤产业占工业比重提高到56%;现代物流、文化旅游等服务业比重提升;农业“一优五特”产业占农业增加值的60%以上。单位GDP综合能耗比“十一五”末下降23.2%。城乡面貌深刻变化。统筹推进以创建全国文明城市为龙头的 “六城联创”，大力开展规范发展煤炭市场工作，建成区绿化覆盖率达到40%，成功创建国家森林城市、园林城市。统筹城乡区域协调发展，沿黄特色小城镇、美丽乡村建设深入推进，城乡一体化进程明显加快，常住人口城镇化率达到74%。人民生活持续改善。每年集中力量办好一批民生实事，基本公共服务水平稳步提升，就业持续扩大，城乡居民收入年均增长超过两位数。累计投入资金130多亿元，实施棚户区改造和保障房建设，建成52877套403万平方米，棚户区改造任务基本完成。搬迁安置移民43887人，移民人均年收入比2010年翻了一番。平安石嘴山建设等工作走在全区前列。发展活力不断释放。全面完成新一轮政府机构改革，新的政务服务中心建成运行，实现审批部门全进驻、审批事项全覆盖，市本级行政审批事项由230项削减为67项。市属国有企业改革、文化行政管理体制改革等取得积极进展，农村土地经营管理制度改革成效显著，形成了全国有影响力的“平罗经验”。

各位代表!过去的五年，我们顶住了压力，经受了考验，虽然增速有所放缓，但结构调整稳步推进，发展质量和效益明显提高，为全面建成小康社会打下了坚实基础。这些成绩的取得，是市委把握大局、正确领导的结果，是市人大、政协加强监督、鼎力支持的结果，是各县区、各部门同心同德、共同努力的结果，凝聚着全市人民的智慧和汗水。在此，我代表市人民政府，向辛勤奋战在全市各条战线的广大干部群众，向各民主党派、工商联、人民团体，向驻石部队、武警官兵、公安干警和驻石中央、区属各单位致以崇高的敬意!向大力支持政府工作的人大代表、政协委员和各界人士表示衷心的感谢!

同时我们也深刻体会到，加快建设开放富裕和谐美丽石嘴山，必须坚定不移地推进工业强市，必须坚定不移地推进“三大转型”，必须坚定不移地深化改革和扩大开放，必须坚定不移地推进依法治市，必须坚定不移地弘扬“五湖四海、自强不息”的石嘴山精神。

在总结成绩的同时，我们更加清醒地认识到，全市经济社会发展还面临不少困难和问题，主要是：经济下行压力较大，部分企业生产经营依然困难，财政收支矛盾日益突出，固定资产投资增长乏力;经济结构不合理，创新驱动发展能力不强，产业转型升级和结构调整任重道远;基本公共服务水平不高，建成全面小康的短板还十分突出，脱贫解困和改善民生的任务艰巨;一些部门和干部执行不力、效率不高、作风不实，依法行政意识和能力有待增强，政府职能还需进一步转变。对此，我们必须高度重视，采取有效措施，认真加以解决。二、“十三五”时期主要任务

“十三五”时期，是我市在全区率先全面建成小康社会的决胜阶段，也是深入推进“三大转型”、建设开放富裕和谐美丽石嘴山的关键时期。党的十八届五中全会为我们指明了发展方向、注入了强大动力，自治区融入“一带一路”、加快开放宁夏建设，为我们带来了新机遇、拓展了新空间。但新常态下，我们面临着扩大总量与提升质量的双重要求，面临着促进增长与保护生态的双重压力，面临着全面小康与脱贫攻坚的双重任务，面临着创新发展与转型追赶的双重考验，不进则退、慢进亦退，机遇稍纵即逝,丝毫松劲懈怠,就会错失良机、贻误发展、落后于人。我们必须增强忧患意识、责任意识和追赶意识，在优化结构上下功夫，在动力转换上求突破，在补齐短板上用实招，努力在新一轮竞争中抢占先机、争取优势、赢得主动，推动经济社会发展迈上新台阶，创造石嘴山成立60周年新成就，让广大群众有更多的获得感和幸福感。

今后五年，我们工作的总体要求是：高举中国特色社会主义伟大旗帜，全面贯彻党的十八大和十八届三中、四中、五中全会精神，以马克思列宁主义、毛泽东思想、邓小平理论、“三个代表”重要思想、科学发展观为指导，深入贯彻落实习近平总书记系列重要讲话精神，主动适应经济发展新常态，坚持“四个全面”战略布局，坚持发展是第一要务，坚持创新、协调、绿色、开放、共享的发展理念，以创新发展、转型追赶为主线，以提高质量和效益为中心，紧紧围绕在全区率先全面建成小康社会目标，坚持开放带动、创新驱动、共建共享、绿色发展，深入推动产业、民生、生态转型发展，加快资源枯竭型城市转型和老工业基地振兴，建设开放富裕和谐美丽新型工业城市。

主要奋斗目标是: 通过创新发展、转型追赶，产业、民生、生态转型取得新成果，开放富裕和谐美丽石嘴山建设迈向更高水平，在全区率先全面建成小康社会。到2020年，地区生产总值年均增长7%左右;固定资产投资年均增长10%以上;一般公共财政预算收入年均增长5%;城镇和农村居民人均可支配收入年均分别增长8%和9%。

实现上述目标，必须牢固树立创新、协调、绿色、开放、共享的五大发展理念，并始终贯穿落实到开放富裕和谐美丽石嘴山建设的全过程，必须牢牢把握石嘴山正处于经济增长换挡期、转型升级关键期和跨越发展机遇期的阶段性特征，在以下五个方面开拓创新，重点突破，合力攻坚：

(一)坚持开放带动，推进共赢发展，构建对外开放新格局。深度融入“一带一路”，积极推进内陆开放型经济试验区建设，着力打造国家级承接产业转移示范区、丝绸之路经济带重要节点城市。借助中阿博览会开放平台，加强与阿拉伯国家和穆斯林地区的交流，深化与约旦亚喀巴经济特区等国外友好城市的合作，争取在丝路沿线和东南亚地区对接一批友好城市;主动寻求与中东部地区的战略合作，有序承接高端产业转移;积极参与呼包银榆经济区、宁夏沿黄经济区建设，推动与毗邻地区基础设施互联互通和资源能源、生态环保、文化旅游等领域合作;加快构筑对外开放通道，向东畅通与天津港海铁联运双向通道，向西推动“宁新欧”铁路班列延伸至惠农陆路口岸，向南连通内陆发达地区，向北推动建设包兰—临哈铁路至乌力吉进出外蒙口岸石嘴山连接线，建设石嘴山沙湖机场，促进城市出口与过境大通道互联互通，构建丝绸之路新通道;加大“三争三引”和“走出去”力度，引进一批大企业、大集团来石投资，扩大优势产品出口贸易规模。发挥宁夏理工学院、宁夏工业学校的人才培养优势，探索建立与丝路沿线国家和地区联合办学机制，着力打造服务本地、面向全国、走出国门的高技能人才培养基地。

(二)突出创新驱动，推进转型发展，培育区域竞争新优势。把创新作为引领发展、转型追赶的第一动力，把产业升级作为主攻方向，下大力气调结构、转方式，推进供给侧结构性改革，着力去产能、去库存、去杠杆、降成本、补短板，加快资源枯竭型城市转型，全力打造石嘴山经济升级版。突出工业提质增效。实施升级改造、增量培育、龙头带动和降本增效“三工程一行动”，构建“4+4”产业集群和特色产业新体系，培育产值达500亿元的产业园区、销售收入达100亿元的企业集团，推进产业向中高端发展。提升服务业发展水平。实施现代物流业提升工程和全域旅游开发工程，建设面向全国和阿拉伯国家的现代物流中心，推进沙湖带动的全域旅游开发;加快发展电子商务、健康养老、社区服务等新业态，推动服务业规模扩大、结构优化、层次提升。到2020年，服务业增加值比重提高到35%以上。提升农业质量效益。实施农业产业化水平提升、农业科技园区示范、耕地质量提升“三个工程”，推进一二三产业融合发展，加快转变农业发展方式，建设宁夏现代农业示范区，促进农业增效、农民增收。实施科技创新行动，大力推进“大众创业、万众创新”，加快创建国家创新型城市，催生新的动能，推动转型升级。

(三)优化发展空间，推进协调发展，实现城乡一体新突破。落实《宁夏空间发展战略规划》，优化市域发展空间，突出“山水园林、重工基地”城市特色，建成宁北并辐射蒙西地区重点城市，提升石嘴山作为宁夏副中心城市的带动力。加快智慧城市、信息惠民城市建设，完善城市治理体系，加强城市精细化管理，创建全国文明城市，让城市和谐宜居更美好。推动城镇公共服务向农村延伸，增强城市和小城镇产业发展、公共服务、吸纳就业、人口集聚功能。加快户籍制度改革和居住证制度双落地，促进有能力在城镇稳定就业和生活的农民举家进城，实现常住人口基本公共服务全覆盖。坚持新村建设和旧村整治并举，加大农村环境整治力度，推进美丽乡村建设，使全市乡镇、村庄都能逐步达到美丽宜居乡村标准。

(四)增进民生福祉，推进共享发展，开创和谐社会新局面。顺应人民群众过上美好生活的新期待，持续加大民生和社会事业投入，让发展成果更多地惠及广大群众。实施居民增收计划，千方百计促进就业创业，持续增加城乡居民收入。实施脱贫攻坚计划，全面完成脱贫攻坚任务，提前在全区消除贫困。实施教育质量提升计划，加快发展学前教育，提高义务教育发展质量，巩固基本普及高中阶段教育成果，努力办好人民满意教育。深化医药卫生体制改革，加强医疗卫生机构重点学科建设，提升城乡医疗卫生服务水平。建立更加公平可持续的社会保障制度，织好民生“安全网”。大力实施文化惠民工程，落实全民健身计划，不断满足群众多样化的精神文化需求。深入开展平安建设和民族团结进步创建活动，维护民族团结、宗教和顺、依法有序、和谐稳定的大好局面。

(五)建设生态文明，推进绿色发展，打造美丽城市新名片。以国家生态文明先行示范区建设为统揽，推动形成绿色发展方式和生活方式，建设宁夏北部生态屏障。加强生态保护与建设，实施贺兰山东麓绿化、绿色通道、防沙治沙等生态绿化工程，到2020年，建成区绿化覆盖率达到42%。实施沙湖—星海湖等水系连通工程，开展重点水域环境综合治理，构建配置合理的水资源管理体系。强化约束性指标管理，开展能源和水资源消耗、建设用地等总量和强度双控行动，全面集约节约和高效利用资源。着力推动绿色循环发展，推进企业、园区循环化改造，实现资源共享、“三废”集中处理与综合利用。加大环境治理力度，深入开展农业面源污染防治，统筹农村饮水安全、垃圾处理。实施环境保护“蓝天、绿水、净土”三项行动，开展贺兰山自然保护区整治，推动贺兰山矿区生态修复治理，有效防治大气、水、土壤污染，持续改善环境质量，让生态文明的城市名片更加靓丽。三、2016年重点工作

2016年是实施“十三五”规划的开局之年，是推进结构性改革的攻坚之年，做好今年各项工作，对“十三五”发展起好步、开好局，具有十分重要的意义。

今年的主要预期目标是：地区生产总值增长7%左右;固定资产投资增长10%左右;一般公共财政预算收入可比增长3%;城镇和农村居民人均可支配收入分别增长8%和9%左右;城镇登记失业率控制在4.3%以内;完成自治区下达的各项约束性指标。

重点抓好七个方面的工作：

(一)注重质量效益提升，推动产业转型升级

全面落实产业转型升级“1+3”方案，打好产业结构调整攻坚战，着力提升经济发展质量和效益。

推进工业转型升级。以优化现有存量、培育优质增量为重点，加快传统产业改造升级，积极培育新兴产业，提质增效促转型，力争规上工业增加值增长7.5% 左右。实施升级改造工程，支持企业瞄准同行业标杆推进技术改造，全面提高工艺装备、产品质量和节能环保水平，重点抓好天地奔牛、英力特化工、滨河碳化硅等企业改造升级，加快推动电石氰胺产业转型发展。实施增量培育工程，发挥产业政策导向和产业发展引导基金作用，鼓励支持中晶光电、恒达纺织、丽珠医药等企业发展壮大，扩张提升新能源、生物医药、新型煤化工、现代纺织产业，提高轻工业比重，以优质增量调存量。实施龙头带动工程，在“4+4”每个产业上培育1— 3家龙头企业，推动大地循环向百亿元企业迈进，促进申银特钢产值超过50亿元、晟晏循环产值超过30亿元。继续推进外地大企业与本地企业、本地企业之间开展重组整合，鼓励支持嘉峰化工、贝利特化工等企业通过产业整合、协作配套等方式做大做强。稳妥化解产能过剩，通过严格环保、能耗、技术标准，倒逼过剩产能退出;引导企业对高污染、高耗能的落后产能实行兼并重组、关停并转;对工艺落后、长期停产、资不抵债、扭亏无望的“僵尸企业”进行清理。

提速第三产业发展。出台《扶持服务业发展的意见》，统筹推动生产性和生活性服务业发展，力争服务业增加值增长6%左右。突出抓好现代物流业。加强同兰州铁路局、甘肃国际陆港公司、天津港的合作，启动中阿物流园建设;完善惠农陆路口岸功能，争取建设保税中心;加快然尔特煤炭铁路专用线、德美斯物流园建设，推动生产性服务业发展。突出抓好文化旅游业。深化与沙湖在旅游规划上的对接融合，抓好奇石山等传统文化旅游景区改造，启动建设“煤城·记忆”景区，做好工业、城市遗迹保护利用，推进大沙湖旅游区建设，吸引游客进市区观光旅游、购物消费。加强旅游从业人员技能培训，完善旅游服务设施，创建国家旅游标准化试点城市。突出抓好消费层次提升。推进汉唐九街、万达广场、汇融·新天地等商贸综合体建设，大力发展线上线下互动，加快商贸流通创新发展和转型升级;实施“互联网+”行动计划，加快大宗工业产品网络在线交易平台建设，推动电子商务、健康养老、家政服务等生活性服务业发展，满足多样化需求，促进消费结构升级;落实房地产去库存的政策措施，把符合条件的存量房转为保障房，鼓励发展住房租赁市场，推动在城市稳定就业的农民工市民化，消化商品房库存，引导房地产业健康发展。

大力发展现代农业。以“一特三高”为引领，培育壮大龙头企业，着力发展精深加工，带动“一优五特”产业发展，力争农业增加值增长5%左右。实施农业产业化水平提升工程，发挥宁羊农牧、泰金种业、益农金禾等龙头企业带动作用，培育各类农村新型经营主体，推进河东地区现代农业示范区建设，打造优质清真牛羊肉生产、优质瓜菜良种繁育等基地。在农业科技园区实施示范工程，建成25个国家级水产健康养殖场、10个高标准现代畜牧业示范场;建成16个标准化露地瓜菜、1.8万亩蔬菜生产基地;优质粮食面积达到55万亩，农作物制种面积达到15万亩，完成国家农业科技园区“一园四区”核心区建设。实施耕地质量提升工程，加大农业基础设施建设投入力度，加强以高效节水为重点的农田水利建设，增强水资源的调控能力，新增节水灌溉面积5万亩、高标准农田2.8万亩，改造中低产田10万亩，提升农业综合生产能力。

(二)紧盯运行关键环节，促进经济稳定增长

认识新常态、适应新常态、引领新常态，沉着应对下行压力，有针对性地采取措施，努力把经济运行稳定在合理区间。

强化项目投资拉动。全力打好项目建设攻坚战，进一步完善领导干部包推项目责任制，设立项目办公室，对新建项目半月一分解、半月一落实，力促各重点项目早开工、早建设、早达效，以项目建设支撑稳增长、促转型。突出抓好90个重点项目，完成投资193亿元以上。重点实施大唐火电、国电大武口热电联产、杉杉能源锂电池正极材料、富海物流二期等产业项目，完成投资117亿元以上;实施银北百万亩盐碱地改良、黄河石嘴山段防洪、大武口森林公园改造、石银高速平罗连接线等农林水、道路交通及基础设施项目，完成投资60亿元以上;实施棚户区改造、养老服务体系等民生社会事业项目，完成投资16亿元以上。提升项目谋划质量和水平，围绕自治区成立60周年等重大活动，主动对接国家重大工程项目包，谋划一批大项目，储备项目600亿元以上，确保持续投资有抓手、长远发展有后劲。

提升招商引资实效。转变招商理念，变“全面招商”为“理性选商”，强化对招商项目投入产出、投资强度、技术装备、环境安全等要素审核，有效防止低水平引进。加大产业招商、企业招商、科技招商力度，主动与国内知名企业开展项目对接，特别是加强与中建材、中煤、中国通用、上海电气等企业合作，争取对接一批新项目、开工一批大项目。重点抓好昊华骏化100万吨乙醇、青纺联织布等42个亿元以上招商项目，力争全年招商引资到位资金增长10%以上。

灵活精准调控运行。全力抓好工业保增长工作，提高经济运行调控水平,逐月逐季分析研判，适时调整全市保增长政策措施，有针对性地开展困难企业帮扶，确保工业企业停产面控制在15%以内，实现全年均衡增长目标。落实降低制度性交易成本、企业税费负担、电力价格、物流成本等政策措施，帮助企业降本提效。在同等条件下，鼓励各类建设项目，特别是政府投资项目，优先采购本地名优产品，有效解决工业产品库存问题。重点保障大地循环、申银特钢、维尔铸造等已投产项目开足马力，多作贡献;力促大生生物、金晶玻璃、盈谷黄金等已开工项目尽快投产，形成增量;鼓励支持有色、煤炭、煤矿机械行业开展内部挖潜，扩大产品销售，抑制减量。加大国家专项建设债券基金、地方政府债券、置换债券的争取力度，缓解企业和政府投资项目资金压力。通过债务置换，将一些短期高成本债务调为长期低成本债务，最大限度降低政府运行成本。加强各类资金监管，提高资金使用效益。强化“两高一剩”行业信贷风险控制，规范各类融资行为，确保不发生系统性和区域性风险。努力做好财政税收工作，大力培植财源，加强税收征管，优化支出结构，盘活存量资金，保障重点支出。加大各类资金争取力度，全年争资金同比增长10%以上。

拓宽项目融资渠道。适应新常态下资本运营的新机制，以产业引导基金、创业创新基金为基础，在纺织、文化旅游等领域设立4个专项子基金;支持中国通用公司等设立发展投资基金，支持台湾宝来公司等设立助贷公司、投资公司，提高信贷灵活性和便利性。搭建国有企业融资平台，扩增鑫鼎担保公司资本金实力，推进与各类金融担保机构的深度合作，进一步扩大面向中小企业的融资担保服务。完善政府风险补偿机制，发挥“助保贷”、“财保贷”作用，解决小微企业融资难的问题。实施PPP示范项目，放大投资效应，引导社会资本投向市政、交通、生态等领域。通过政策引导、以奖代补等方式,支持符合条件的优质企业在股权和债权市场融资，推动4家企业挂牌交易。

(三)落实空间发展规划，推进城乡协调发展

以空间发展战略规划为引领，推动以人为核心的新型城镇化，促进城镇公共服务向农村延伸，加快城乡一体化进程。

科学编制发展规划。推进“多规合一”，对城市建设总体规划、土地利用总体规划和重点区域规划进行修编完善，形成规划“一张图”，加强规划监督执行，切实维护规划的严肃性和权威性。落实市委“十三五”规划建议，编制好全市“十三五”发展规划纲要及产业发展、基础设施、生态环保等专项规划。统筹做好村镇建设规划、美丽乡村规划及城市地下综合管廊、水系连通改造、中阿物流园、全域旅游开发规划编制，优化城乡发展布局。

完善城镇服务功能。加强城市地下和地上基础设施建设，完善供排水、燃气、供热、垃圾处理等基础设施，完成第二污水处理厂扩容升级及第五污水处理厂建设，争取启动建设城市地下综合管廊。优先发展城市公共交通，面对公交私营的亏损局面，国有企业要主动担当，承担应尽的社会责任和义务，实施公交公营，加快同城化步伐。合理布局建设停车场、公交站亭及免费公厕，方便群众生活。加快“智慧城市”建设，90%以上的城市家庭具备光纤接入能力，80%以上的行政村实现光纤到村，争创信息惠民示范城市。实施城市亮化美化工程，提升城市品位。建成棚改安置房2603套、公共租赁住房2941套，完成2个美丽小城镇、8 个美丽乡村建设。

提高城市管理水平。全面推进数字化城市管理，完善市级和县区数字化城管系统平台，推行“大城管”综合执法模式，引导市民共同参与城市管理。推进地方立法进程,确保行政执法有法可依。深入开展公共环境、公共秩序整治，加大整治城市建成区私搭乱建现象力度，加强各类专业市场、早市夜市的管理。稳步推进环卫作业专业化、市场化，提高机械化清扫率。出台《物业规范管理监督办法》，继续开展规范化物业小区创建，扩大城市区老旧小区、移民小区物业管理覆盖率。

统筹推进“六城联创”。在巩固国家园林城市、森林城市创建成果的基础上，深化各类文明创建活动，启动国家节水型城市、中国人居环境奖创建工作，全力争创全国双拥模范城市、卫生城市、法治城市、社会管理综合治理优秀城市、残疾人工作先进单位，为成功创建全国文明城市奠定坚实基础。(四)推进生态文明建设，着力改善环境质量

把生态文明建设融入经济社会发展全过程，加强生态治理和环境保护，推进绿色、循环、低碳发展。

加大生态建设力度。推动贺兰山自然保护区规划调整。坚持扩大规模、提升档次、打造精品，重点实施城区景观绿化改造提升、绿色通道建设、高标准农田林网等绿化工程，推进开发区和居民小区绿化，继续完善市民休闲森林公园建设，造林2.5万亩。加快水生态文明城市建设，启动实施沙湖—星海湖水系连通工程，推动建立跨市县重点流域和区域水污染治理机制，开展水环境联防联治，实施沙湖、星海湖水质富营养化防控，做好主要排水沟黑臭水体生态治理。严格水资源开发利用管理，加强饮用水源地保护，关停20眼自备井。

大力发展循环经济。加快国家循环经济示范城市、国家资源综合利用“双百工程”示范基地建设，推动石嘴山经开区、生态区循环化改造，实施67个循环化改造项目，推进昌茂祥、惠义冶金等5家企业烟气余热综合利用，形成企业间和园区间废弃物交换利用、能量梯级利用的循环产业体系。重点抓好日盛化工、吉元冶金等一批循环链增补项目，实施中煤集团低质煤提级等循环经济示范项目，促进企业循环式生产、产业循环式组合。

强化环境污染防治。严格环保准入和污染物排放标准，切实加大环境监管力度，重拳惩治环境违法行为。实施大气污染防治行动计划，加强工业园区、城乡结合部等重点区域、重点污染源综合治理，对2家自备电厂发电机组和14台20蒸吨以上燃煤锅炉进行污染治理提标改造，淘汰取缔燃煤小锅炉。完善煤炭集中区基础设施配套，巩固提升规范发展煤炭市场成果。持续推进规模化畜禽养殖企业污染治理，推进秸秆禁烧和综合利用，力争PM2.5下降3%、PM10下降5%。

(五)加强区域交流合作，提升对外开放水平

坚持引进来和走出去并重，全面开放与重点突破结合，发展更高层次的开放型经济，不断扩大对内对外开放。

扩大区域合作。联合自治区商务厅在迪拜召开中阿物流产业发展论坛，深化与国外友好城市交流合作，支持有条件的企业抱团赴外投资、拓展市场。加强与京津冀、长三角等地区的联系对接，引进和承接产业转移项目。落实与乌海、鄂尔多斯等周边城市合作协议，在交通基础设施建设、环境污染防治等方面开展务实合作。推进与宁夏农垦集团的交流，协商土地参股共建园区新模式。逐步推动市场化举办节会，提高本地群众的参与度，吸引更多外地人员参与，继续办好国际铁人三项赛、国际标准舞公开赛、全国室内五人制足球赛、全国露营大会等活动，打造特色节会品牌。

构筑开放通道。以被列入国家66个区域流通节点城市和兰州铁路局7个区域性物流中心城市为契机，发挥交通枢纽优势，加快推进红崖子黄河公路大桥、国道 244线高仁至宁蒙界、国道110线正谊关至宁蒙界、省道301线太西镇至宁蒙界等“一桥四路”建设，争取启动建设京藏高速石嘴山段改扩建工程，做好向北开放通道项目的论证工作。依托惠农陆路口岸，开行中欧班列“石嘴山号”。

优化开放平台。实施开放强园工程，进一步完善开发区道路、供排水、天然气等基础设施，完成石嘴山经开区、宁夏精细化工基地污水处理厂建设，推进石嘴山生态区、高新区污水处理厂和固废处置场建设，新建宁夏精细化工基地黄河水厂。打造水电路配套、土地集约利用、要素功能聚集的低成本园区，为入园企业降低成本提供保障。加快实施开发区输电线路改造，加大闲置土地清理力度，采取有效措施拓展发展空间。围绕开发区的不同定位，建设淄山工业园、电子材料产业园、智能硬件产业园等各具特色的专业园中园，促进产业做大做强。

(六)深入推进改革创新，增强发展内生动力

坚持把改革创新贯穿于经济社会发展各领域，以改革创新破解发展难题、增强发展活力、推动持续发展。

推进重点领域改革。扎实开展全国中小城市综合改革试点，着力解决制约发展的深层次问题。深化行政审批制度改革，进一步简政放权，在上半年公布政府部门权力清单、责任清单，对已经明确取消、转移、下放的行政审批事项，抓好落实、承接工作，对现有行政审批事项进行再清理，该减的减，该放的放。深化市属国有企业改革，落实国有企业薪酬改革办法，提高国有资产监管、国有资本运行水平和效率。推进户籍制度改革，逐步实现转移人口享受同城同等待遇。加快推进农村改革，推动农村土地承包经营权和流转经营权抵押贷款“扩面增量”，抓好平罗县全国农村产权流转交易市场建设、农村宅基地制度改革试点。做好公务用车制度改革，按照自治区批复要求，把握关键，稳妥推进，第一季度基本完成改革任务。

推进小微企业“双创”。加快建设宁夏智能硬件产业园、启明星众创空间等，提升各开发区创业孵化园、大学生创业孵化园服务功能，努力打造西北地区乃至全国创业创新亮点。探索建立“创业、创新服务券”新模式，落实扶持小微企业政策措施，新增小微企业1000余户。大力培育创新主体和创业主体，实施西部青年创业创新项目，做好大学生、复转军人、残疾人、城镇失业人员创业培训指导，新增就业2.1万人。

提高自主创新能力。加大科技投入力度，建立和完善企业科技创新后补助机制，支持新兴产业、现代服务业、现代农业等领域的科技创新活动。强化企业科技创新主体地位和主导作用，支持企业加强与国内外科研院所、高校、知名企业开展科技合作，共建重点实验室、技术创新中心等创新平台，不断提高自主创新能力。启动科技“育龙工程”，鼓励支持科技型中小企业开展产品创新、品牌创新，形成一批具有竞争力的创新型企业。强化“三争三引”，千方百计引进和培养高端人才、产业领军人才、技术创新人才，支撑产业发展。

(七)加快发展社会事业，推进发展成果共享

以增进人民福祉为目的，增加民生投入，兜底线、补短板、促公平，提高公共服务共建能力和共享水平。

大力发展教育卫生事业。统筹城乡教育资源配置，完成市星海中学、青少年示范性综合实践基地建设，开工建设市实验幼儿园第三分园。提升教育质量，加强乡村教师队伍建设，缩小城乡、校际差距，让优质教育惠及更多家庭。深化产教融合、校企合作，畅通学生从中职到高职、从高职到应用型本科的教育渠道，加快建设宁夏高技能人才培养基地。推进公立医院改革，探索建立医疗风险共担机制，实行医疗、医保、医药联动。进一步健全基层医疗卫生机构，建成市儿童医院及妇幼保健院。稳步推进职工医保门诊统筹、城乡居民大病保险和普惠性健康体检等工作，更好满足群众健康需求。建立村医准入、退出和收入补偿机制，完善村医养老保障。巩固提升幸福家庭创建成果，落实一对夫妇可生育两个孩子政策。

繁荣发展文化体育事业。完成国家公共文化服务体系示范区创建，推进智慧公共文化系统工程，整合图书、影视、非遗等文化资源，建设公共数字文化服务云平台。加大具有地域特色的文艺精品创作力度，扶持非营利民间艺术团发展，提升文化服务供给能力。办好三大文化品牌活动，年内完成200场广场文化演出、 200场“送戏下乡”演出。扩大文化体育资源向市民免费开放范围，开展形式多样的全民健身活动，办好各类体育赛事，丰富广大群众特别是老年人的精神文化生活。

健全完善社会保障体系。推进全民参保登记，稳步实施 “五险合一”，加快推进机关事业单位养老保险并轨，抓好失地农民参加养老保险工作。支持民营资本兴办养老服务机构，建设市敬老院、老年活动中心，完善城乡社区居家养老服务站功能;推动医疗卫生和养老服务相结合，提高养老服务业发展水平。建成残疾人康复中心。完善各类社会救助体系，让更多的困难群众求助有门、受助及时。

扎实做好扶贫脱贫工作。实施脱贫攻坚计划，全面摸清全市贫困人口基本状况，准确掌握贫困人口底数，对贫困人口逐一建档立卡。坚持精准扶贫、精准脱贫，建立脱贫长效机制，分类扶持贫困家庭，扎实推进基础设施到村、项目扶持到户、培训转移到人、帮扶责任到单位的“四到”扶贫攻坚，对有劳动能力的支持发展特色种养、商贸运输、劳务等产业，对因病致贫的提供医疗救助保障。

加强和创新社会治理。深化平安石嘴山建设，构建立体化治安防控体系，深入开展新一轮禁毒人民战争。启动“七五”普法，完善矛盾纠纷多元化解机制，加大法律援助力度。坚持就地解决信访问题，积极做好信访积案化解，切实维护群众合法权益。依法管理民族宗教事务。加强应急管理，进一步提高公共安全、防灾、减灾和救灾能力，及时预防和处置群体性、突发性公共事件。全面落实安全生产责任制，强化道路交通、煤矿及非煤矿山、危化企业、食品药品安全监管，坚决遏制重特大安全事故发生，确保群众安居乐业、社会安定有序。

坚持尽力而为、量力而行原则，继续办好10件民生实事，让群众得到更多的实惠。1.加大创业和电商培训力度，建设大学生网上创业体验中心，继续培训新型职业农民1800人。2.建设公共自行车租赁点，实施自行车慢行交通系统工程。3.为困难群众免费办理法律援助案件700件，人民调解组织化解各类矛盾纠纷4000件。4.在城市主次干道、商业大街建设无障碍设施3.74万平方米，对69座室外公共厕所进行无障碍设施改造，整治无障碍设施被占用现象。 5.新建4G基站150处，全市4G网络覆盖率提升至95%以上，全面推进宽带网络提速降费。6.完成老旧住宅小区节能改造71万平方米。7.在市区增设 1960个免费停车泊位。8.实施全民健身计划，建成3个体育主题公园。9.淘汰取缔100台燃煤锅炉。10.新建一所敬老院，进一步扩大敬老养老能力。四、切实加强政府自身建设

各位代表，新形势、新任务，对政府工作提出了新的更高的要求。我们将牢记使命，不负重托，用恪尽职守诠释责任，用奋发有为回应期待，以更高的标准、更严的要求，进一步加强自身建设，努力建设人民满意政府。

(一)完善机制，科学决策，全面推进依法行政。贯彻落实国家《法治政府建设实施纲要》、自治区《法治政府建设指标体系》，严格按照法定权限和程序行使权力、履行职责。更好地发挥市政府法律顾问团的决策参谋作用，推进政府依法、科学、民主决策。加强电子政务建设，推进政府信息公开，提高政府工作透明度。认真办理人大代表建议和政协委员提案，继续开展建议和提案办理后评估，促进办理质量提升，提高群众满意率。自觉接受人大及其常委会的法律监督、工作监督和政协的民主监督，加强与各民主党派、工商联、人民团体和无党派人士的联系，充分尊重和发挥其参政议政作用。

(二)改进作风，主动作为，着力提高行政效能。以提高政府执行力为重点，继续完善和深入推行市级领导“双包”、部门季度考评、电视问政、干事档案等监督机制，完善自上而下、逐级负责的责任体系，切实解决落实不力、不实的问题。加强对重大决策、重要工作、重点项目的督促检查，加大问责力度，严肃惩治慢作为、不作为、乱作为的人和事。优化政务服务中心运行管理，找差距、提质量、树亮点，拓展完善服务功能;定标准、强管理、抓效率，提升服务水平;加强硬软件建设，推动管理服务信息化，为群众和企业提供更便捷、更高效的服务。大兴求真务实之风，以踏石留印、抓铁有痕的劲头抓工作，不折不扣落实国家、自治区出台的各项惠民政策，说到的就要做到，承诺的就要兑现，努力做出经得起实践、历史、人民检验的实绩。

(三)从严治政，惩防并举，切实做到清正廉洁。为政清廉才能取信于民，秉公用权才能赢得人心。严格落实党风廉政建设责任制，发挥好监察、审计、司法等职能部门作用，加大对行政审批、工程建设、土地出让、政府采购等重点领域的监管力度，着力解决损害群众利益的突出问题，严厉查处违纪违法行为。认真落实 “八项规定” 精神，牢固树立过紧日子的思想，坚持厉行节约、精打细算，从严控制行政经费和一般性开支，真正把财政资金用到发展最需要、群众最受益的地方。各级政府和广大干部要倍加珍惜党和人民赋予的权力，自觉遵守《廉洁自律准则》和《纪律处分条例》，践行“三严三实”，清清白白做人，干干净净做事，扎扎实实创业，树立为民务实清廉的良好形象。

各位代表，时代赋予重任，奋斗铸就辉煌。让我们在市委的坚强领导下，进一步坚定信心，开拓创新，锐意进取，务实苦干，为加快推进开放富裕和谐美丽石嘴山建设，在全区率先全面建成小康社会而努力奋斗!
